# Infrastructure Preparedness for Autonomous Vehicles in Rural Areas

**DOI:** 10.3390/s26196067

**Published:** 2026-09-24

**Authors:** Joshua X. S. Li, Benjamin X. Y. Li, Yiming Yang, Junlong Liu, Joshua Q. Li, Anirudh Paranjothi

**Affiliations:** 1 Oklahoma School of Science and Mathematics (OSSM), Oklahoma City, OK 74074, USA; lijoshua0121@gmail.com; 2 Stillwater High School, Stillwater, OK 74075, USA; libenjamin100@gmail.com; 3 School of Civil Engineering, Oklahoma State University, Stillwater, OK 74078, USA; junlong.liu@okstate.edu (J.L.); qiang.li@okstate.edu (J.Q.L.); 4 Department of Computer Science, Oklahoma State University, Stillwater, OK 74078, USA; anirudh.paranjothi@okstate.edu

**Keywords:** infrastructure preparedness, autonomous vehicle readiness index, rural transportation, analytic hierarchy process

## Abstract

While advancements in autonomous vehicle (AV) technology heavily favor urban environments, rural and tribal regions remain severely underprepared for automated deployment due to physical infrastructure deficiencies and communication limitations. To address this disparity, this paper formulates a scalable, low-cost multimodal framework designed to systematically evaluate rural infrastructure readiness across three critical operational domains: communication connectivity, lane marking detectability, and road surface kinematics. First, cellular network reliability is mapped via mobile telemetry to isolate connectivity-deficient risk zones. Second, a vision-based perception module leverages deep learning models on GPS-tagged video footage to quantify lane marking detectability. Third, an onboard road surface assessment pipeline captures inertial measurement unit (IMU) and GPS data to calculate safety-critical geometry metrics—including rollover risk and composite acceleration—while introducing a rapid estimation using the Analytic Hierarchy Process (AHP) of the roadway International Roughness Index (IRI). These sub-indicators are weighted and synthesized into a unified Autonomous Vehicle Readiness Index (ARI) to map to the spatial readiness of AV-critical infrastructure systems. Validated across underserved rural corridors, this framework provides transportation agencies with an objective, data-driven decision engine to prioritize localized infrastructure remediation and accelerate safe, equitable AV deployment.

## 1. Introduction

The future of transportation is rapidly evolving with the advancement of Autonomous Vehicles (AVs), which promise improved road safety, reduced traffic congestion, and enhanced mobility. However, the success of full-scale AV deployment relies heavily on reliable and well-maintained transportation infrastructure [1]. This need becomes even more important in rural and tribal areas, where infrastructure is often sparse, under-maintained, or inconsistently developed compared to urban areas, due to low population density, fewer vehicle miles traveled, and limited funding [2]. For AVs to function reliably in these regions, foundational infrastructure elements such as uninterrupted connectivity, clear lane markings, satisfactory conditions, and a safe vehicle manner must be ensured [3]. Based on the existing literature, this study develops a data-driven framework for the infrastructure preparedness of rural road segments focusing on three key components: (1) cellular signal strength monitoring of real-time Long-Term Evolution/5th Generation Wireless Networks (LTE/5G), (2) detection and evaluation of lane marking using deep learning-based detectors, and (3) assessment of roadway surface conditions using both dedicated testing measurements and model-based estimation for fast evaluation. These efforts aim to identify gaps and provide insights that can assist transportation agencies in making rural roadways more AV-ready.

Cellular signal strength plays a crucial role in maintaining uninterrupted communication between AVs and the surrounding transportation infrastructure [4]. This connectivity supports essential operations, including Vehicle-to-Everything (V2X) communication for Connected and Autonomous Vehicles (CAVs), cloud-edge data exchanges, and Over-The-Air (OTA) system updates [5]. In this study, we evaluated spatial variations in LTE/5G signal strength across real-world travel routes using a mobile monitoring framework. Signal strength metrics were recorded alongside GPS coordinates via a smartphone logging application, enabling segment-level performance analysis.

Assessing lane marking quality represents a critical component of evaluating infrastructure readiness for AV deployment, particularly in rural environments. Computer vision systems onboard AVs rely heavily on well-defined and consistently placed lane markings for accurate lane-keeping, navigation, and safe maneuverability [6]. In this study, GPS-tagged video footage of selected rural routes was captured using a GoPro Hero 13 action camera. The recorded video was processed through a deep learning-based lane detection neural network that assigned confidence scores to both left and right lane boundaries. This module enabled the spatial identification of faded, degraded, or missing markings that pose operational challenges for AV perception systems.

Finally, roadway geometry and surface condition are critical determinants of safe AV operation [7,8]. Effective road condition monitoring essentializes both timely infrastructure maintenance and seamless AV deployment. Although traditional assessment methods—such as inertial profilers and 3D laser imaging platforms—provide high-fidelity data, their elevated operational costs and multi-year survey cycles restrict their application primarily to high-tier highway networks [9,10]. Consequently, these capital-intensive approaches lack the real-time scalability required for continuous AV operational support. To address these limitations, this study presents a low-cost, scalable framework that leverages onboard IMU and GPS data to estimate IRI. Field telemetry collected from a 3D highway vehicle was utilized to validate the proposed fast estimation approach. Other key performance parameters, including rollover risk and composite acceleration were also calculated to evaluate the safety-critical indicators of operating vehicles.

To establish appropriate data quality and precision requirements, the target operational purpose of the proposed assessment must be clearly defined. Rather than serving as a real-time vehicle control loop—which demands sub-centimeter survey precision and high-frequency sensor streams—the primary purpose of the ARI framework is proactive, agency-level asset management and corridor screening. According to the infrastructure preparedness literature and Infrastructure Support for Automated Driving (ISAD) concepts [11], decision-support tools for public agencies require a “fit-for-purpose” data quality threshold. Specifically, the assessment quality must provide sufficient spatial resolution (e.g., 0.01mile) to isolate localized physical and digital ODD deficiencies (e.g., faded lane markings or terrain-induced cellular drops) while maintaining low computational and data-collection costs suitable for network-wide deployment [12].

The remainder of this paper is organized as follows: Section 2 reviews the existing literature on infrastructure readiness, highlights current research gaps, and outlines the primary objectives of this study. Section 3 presents the proposed system architecture and infrastructure preparedness evaluation pipeline. Section 4 details the implementation setup, including rural route selection, hardware configurations, and field data collection procedures. Section 5 describes the data processing methodology and experimental results. Section 6 presents the aggregated evaluation results for infrastructure roadway evaluation, using an Analytic Hierarchy Process (AHP) survey to derive weightings for the three primary evaluation metrics. Finally, Section 7 concludes the paper and highlights potential avenues for future research.

## 2. Literature Review

The mass deployment of AVs and CAVs is anticipated to transform urban mobility by 2030, necessitating a comprehensive reassessment of existing road infrastructure for autonomous driving compatibility. This transition is guided by international standards, most notably the SAE J3016 taxonomy [13], which defines six levels of driving automation ranging from no automation to full automation. While global indices like the KPMG Autonomous Vehicles Readiness Index [14] have provided high-level national benchmarks focusing on policy, technology, and consumer acceptance, researchers have increasingly sought more localized, quantitative metrics to evaluate readiness at the city, corridor, and rural levels. The development of structured readiness assessments began in earnest around 2019. Khan et al. [15] proposed the first urban readiness index, identifying policy, physical infrastructure, and cyber infrastructure as three pillars for facilitating CAV deployment. Simultaneously, Saeed et al. [16] addressed the inherent uncertainties of AV market penetration by applying Real Options Analysis to justify phased infrastructure retrofitting, emphasizing that the benefits of AVs remain latent until infrastructure is sufficiently prepared.

As the field matured, studies moved toward closing the gap between traditional roadway design and the specific sensory needs of CAVs. Gouda [17] introduced raycasting approaches to assess how road geometry affects an AV’s ability to “see the road” through its sensors. Jiang et al. [18] further refined city-level assessments by utilizing AHP to weight criteria based on multi-stakeholder perspectives, finding that professionals and the public prioritize infrastructure quality differently.

Consumer and psychological readiness also emerged as critical dimensions. O’hern et al. [19] utilized the Technology Readiness Index (TRI 2.0) to explore how optimism and insecurity influence intentions to use automated vehicles, noting that prior exposure to driver assistance features significantly increases user trust. In the following work, Bilici expanded this by examining the role of anthropomorphism in AV technology acceptance, discovering that human-like AI features can mitigate consumer discomfort and distrust [20]. Regional analyses, such as the neural network approach applied to Hungarian cities have identified that a city’s internal area and business density are strong predictors of AV testing expectations [21].

Most recently, research has pivoted toward operational action plans and granular corridor-level diagnostics. Toth et al. [22] developed a five-year strategic action plan for the Tennessee Department of Transportation (TDOT), highlighting gaps in skilled personnel and the need for clear regulatory frameworks. Pillala et al. [23] integrated cost–benefit analysis (CBA) with technology readiness levels to provide a decision-support tool for highway operators to prioritize economically viable infrastructure modifications. At the most localized scale, Kiattikomol et al. [24] conducted field-based assessments of Bangkok’s controlled-access roads, identifying specific deficiencies in 5G network speeds and lane marking visibility. For rural areas, which have been historically neglected, a framework has been developed using secondary data to assess digital and physical infrastructure, identifying Camille County, North Carolina, as a prime candidate for early automated shuttle deployment due to its broadband availability [25]. Finally, a quantitative Highway Readiness Index (HRI) was proposed to map static Operational Design Domain (ODD) conditions—such as signage quality and high-definition maps—directly to specific SAE levels of automation [26].

Despite these advancements, the existing literature reveals critical methodological gaps when evaluating rural AV infrastructure preparedness, as summarized in Table 1. First, state-of-the-art indices predominantly rely on either static, macro-level secondary datasets (e.g., county broadband maps or HPMS database records) or costly, specialized survey vehicles restricted to urban highways. Second, physical surface metrics (e.g., pavement roughness), perception enablers (e.g., lane marking visibility), and digital connectivity (e.g., cellular signal strength) are frequently evaluated in isolation rather than synthesized into an integrated operational metric. Third, dynamic roadway kinematics—such as lateral rollover risk and dynamic vehicle accelerations—are consistently omitted from infrastructure readiness assessments, despite their critical impact on rural AV control stability.

To bridge these gaps, this study introduces four primary contributions to the field of AV infrastructure assessment:

1. Low-Cost Multi-Sensor Sensing Model: Replaces expensive dedicated survey profiling rigs with a low-cost onboard sensor suite (camera, GNSS/IMU, and mobile telemetry) capable of rapid, scalable deployment by local transportation agencies.
2. Integrated Physical–Digital Framework: Simultaneously evaluates physical surface dynamics, visual perception detectability via deep learning, and dynamic digital connectivity within a single unified index.
3. Incorporation of Dynamic Kinematics: Introduces the rollover risk index (RRI) and composite acceleration (CA) metrics alongside a rapid AHP-estimated IRI to capture vehicle–road operational safety.
4. Micro-Scale Spatial Resolution: Evaluates infrastructure readiness on a high-resolution 0.01-mile (∼52.8 ft) grid, capturing localized surface anomalies and signal dead zones masked by traditional macro-scale agency reporting.

To achieve these contributions, the primary objective of this study is to assess rural infrastructure preparedness for AV deployment using scalable, cost-effective sensing methods. To achieve this goal, the study includes the following specific research objectives:

- Evaluate LTE/5G signal coverage along selected rural corridors and establish a scalable framework to map critical communication gaps.
- Assess lane marking visibility and degradation by integrating video logs with deep learning-based detection models.
- Develop and validate a low-cost IRI prediction model for pavement condition monitoring, bypassing the need for dedicated profiling equipment.
- Estimate key roadway geometric and safety parameters, specifically rollover risk and composite acceleration, from onboard sensors.
- Synthesize and aggregate multi-source evaluation metrics into a unified index to quantify comprehensive AV infrastructure readiness.

## 3. Proposed Methodology

This section presents the comprehensive methodology developed to evaluate the proposed infrastructure preparedness framework for AVs in rural environments. The framework integrates cellular signal evaluation recorded via a smartphone running G-NetTrack Pro v19.6, lane marking detection captured with a GoPro HERO13 Black camera (GoPro, Inc., San Mateo, CA, USA), and roadway condition assessment measured with an AMES Profiler system (Ames Engineering, Ames, IA, USA) into a unified workflow. This methodology is structured to ensure cost-efficiency, reproducibility, and engineering interpretability across all evaluation stages. As depicted in Figure 1, the system architecture forms an end-to-end evaluation pipeline encompassing hardware, data processing, AHP-based weight calculation, and metric aggregation.

### 3.1. Hardware and Software Configuration

To ensure operational reproducibility and clarify the low-cost nature of the proposed framework, Table 2 provides a detailed breakdown of hardware components, sensor specifications, software platforms, and data processing tools used across the existing research. Furthermore, the table contrasts our onboard deployment suite with the hardware/software architectures reported in the recent AV readiness literature.

### 3.2. Domain Evaluation and Processing

The raw field data is processed through domain-specific workflows: (1) signal strength evaluation: which computes spatial signal statistics and coverage performance; (2) lane marking evaluation: which executes deep learning-based computer vision routines to detect left-lane and right-lane markings and evaluate the output confidence values; (3) road condition assessment: which extracts pavement condition and roadway safety metrics, including IRI, rollover risk index, and composite acceleration.

### 3.3. Weighting and Aggregation

To translate raw and separate engineering metrics into actionable decision-making weighting factors, subject-matter expert preferences are gathered using an AHP survey. The resulting relative weights are applied to aggregate the individual domain metrics into a unified ARI.

## 4. Implementation

### 4.1. Rural Route Selection

To ensure a representative field dataset, two distinct rural corridors within a two-hour driving radius of the Oklahoma State University (OSU) campus were selected for framework evaluation (Figure 2). Route 1 (92.6 miles), located between Oklahoma City and Tulsa, OK, was designated for model development, calibration, and validation across diverse geometric curvatures, elevation changes, pavement states (new asphalt, degraded surfaces, and unpaved gravel), and lane marking conditions (dual-sided, single-sided, or absent). Route 2 (52.4 miles), situated southwest of Stillwater, OK, served as an independent deployment testbed to evaluate generalized model performance.

Model training and validation were conducted at a 0.1-mile spatial resolution on route 1 to align with common DOT standards for pavement management purposes. In contrast, the finer 0.01-mile interval was chosen for the final ARI mapping to provide greater spatial granularity and capture localized infrastructure anomalies (such as abrupt joint distress or severe localized roughness) that may directly affect AV operational safety and ODD thresholds on route 2.

### 4.2. Hardware Configuration

The experimental setup integrated three sensing modalities onto a unified mobile test platform, as illustrated in Figure 3: (1) a cellular logging unit that uses a smartphone equipped with a dedicated signal logging application recorded real-time LTE/5G signal strength along the travel routes; (2) a visual perception unit that utilizes a GPS-enabled GoPro Hero 13 action camera captured video logs of the roadway surface and lane boundaries; and (3) a high-speed 3D highway vehicle equipped with high-precision inertial profilers, 3D laser imaging sensors, high-grade IMU/GPS navigation units, and a Distance Measuring Instrument (DMI) was deployed simultaneously to collect roadway roughness and geometric data.

### 4.3. Data Acquisition

Telemetry was collected across both selected routes under normal driving conditions while adhering to posted speed limits. Because the heterogeneous sensor suite recorded data across varying sampling frequencies and native file formats, a spatiotemporal synchronization pipeline was executed. Spatial alignment was achieved using GPS coordinates collected across all three sub-systems, anchored by the high-precision DMI wheel encoder measurements from the 3D profiling vehicle as the spatial baseline.

To accommodate the differing analytical objectives of the two field tests, the multi-modal measurements were spatially resampled using route-specific aggregation intervals. For route 1, data was aggregated into 0.1-mile (approx. 160.9 m) segments to provide a rapid, macroscopic demonstration of the overall analytical framework. Conversely, for route 2, a finer 0.01-mile (approx. 16.1 m) interval was utilized to achieve higher spatial resolution, enabling a highly granular evaluation of localized pavement anomalies and signal variations. For spatial segments containing multiple data samples, the raw values and their corresponding GPS coordinates were averaged to derive a single, synchronized representative metric per interval.

To isolate intrinsic road-induced excitations from vehicle and driver variability, field data collection adhered to a standardized experimental protocol. Telemetry was gathered using a single, baseline probe vehicle (Figure 3) with fixed kinematic properties (mass distribution, tire pressure, and suspension configuration) operated by a single driver maintaining posted speed limits under steady-state lane-keeping conditions. Synchronized GPS and IMU profiles were screened to filter out transient non-infrastructure maneuvers—such as sudden braking, aggressive steering, or intersection stops—prior to dataset processing. Furthermore, dynamic metrics (CA and RRI) were velocity-normalized to ensure that spatial variations reflect intrinsic roadway surface roughness and cross-slope geometry rather than speed discrepancies.

### 4.4. Indicator Normalization

To synthesize heterogeneous metrics into the unified ARI framework, each indicator is normalized onto a non-dimensional readiness scale bounded within [0,1], where 1.0 represents optimal AV infrastructure readiness and 0.0 represents severe operational risk. Indicators are categorized based on their functional directionality: *Benefit* criteria (where higher raw values indicate higher readiness) and *Cost* criteria (where lower raw values indicate higher readiness). Min–max linear scaling is applied according to Equations (1) and (2):

(1) $$x_{\mathrm{norm}}=\frac{x-x_{\min}}{x_{\max}-x_{\min}}\;(\mathrm{Benefit}\;\mathrm{Criteria}:\;\mathrm{Signal}\;\mathrm{Strength},\;\mathrm{Lane}\;\mathrm{Detection})$$

(2) $$x_{\mathrm{norm}}=\frac{x_{\max}-x}{x_{\max}-x_{\min}}\;(\mathrm{Cos}t\;\mathrm{Criteria}:\;\mathrm{IRI},\;\mathrm{RRI},\;\mathrm{Composite}\;\mathrm{Acceleration})$$

where *x* is the observed raw value, and $x_{\min}$ and $x_{\max}$ denote the operational baseline bounds defined in Table 3. To prevent momentary physical anomalies (e.g., severe sensor jolts or temporary telemetry drops) from distorting the scaling, extreme outliers were clipped at the 1st and 99th percentile physical boundaries prior to normalization. Isolated missing spatial records (<0.05 miles) were imputed via spatial linear interpolation using adjacent frame averages, whereas contiguous outages exceeding 0.05 miles were flagged and excluded from index calculation.

## 5. Field Data Analysis and Results

### 5.1. Signal Strength Evaluation

#### 5.1.1. Signal Quality Assessment

For both route 1 and route 2, the signal strength observations were categorized into four quality classes: Excellent, Good, Fair, and Poor, based on established thresholds, as detailed in Table 4. For route 1, the vast majority of observations fell into the Poor category (N=792), followed by Fair (N=102), Good (N=30), and Excellent (N=2). For route 2, out of 5237 evaluated 0.01-mile intervals, 3546 (67.7%) were characterized by Poor signal coverage. Fair coverage accounted for 6.25 (11.9%), Good coverage was observed in 6.07 segments (11.6%), and Excellent coverage occurred in only 456 segments (8.8%). Combined, 79.6% of route 2 exhibited Poor or Fair cellular connectivity.

#### 5.1.2. Signal Strength Distribution

Figure 4 illustrates the macroscopic distribution of the average Reference Signal Received Power (RSRP) across the evaluated route 2. As depicted in the histogram (Figure 4a), the network connectivity exhibits a severe negative skew, with the vast majority of spatial intervals falling into the Poor category (below −100 dBm). While the system did record sporadic intervals of Good and Excellent connectivity—reaching peak signal strengths near −56 dBm—these instances were highly transient. This imbalance is further quantified by the empirical Cumulative Distribution Function (CDF) in Figure 4b, which demonstrates that approximately 67.7% of the total evaluated route operated under Poor signal conditions, presenting significant challenges for high-bandwidth data transmission. Furthermore, as observed in the empirical CDF, a prominent vertical step occurs near −104 dBm, corresponding to the towering frequency peak in the adjacent histogram. This artifact is a direct result of the spatial linear interpolation applied across short, isolated cellular telemetry gaps (<0.05 miles). Ultimately, these combined distributions emphasize the severity of localized coverage gaps and underscore the necessity of robust edge-buffering protocols for continuous autonomous vehicle operations in non-ideal network environments.

Figure 5 provides a dual-perspective analysis of cellular connectivity and topographic correlation across the test route. Figure 5a depicts the spatial signal heatmap, highlighting distinct geographic clusters of high signal strength (green) along the northwest and southwest track segments, alongside severe coverage degradation (orange and red) along the western and eastern corridors. Figure 5b correlates these trajectory observations with local terrain elevation (m) using a moving average trend. The profile reveals a strong, non-linear relationship between topography and cellular propagation: at mile 15, a steep elevation drop to approximately 235 m coincides with severe signal degradation down to −125 dBm, likely attributable to terrain shadowing. Conversely, local elevation peaks around miles 21 and 34 provide unobstructed line-of-sight to nearby base stations, producing optimal signal levels near −60 dBm. Notably, between miles 26 and 30, high elevation coincides with degraded signal strength (below −110 dBm), suggesting that cellular performance along this corridor is governed not by elevation alone, but likely by a combination of terrain geometry and unmeasured localized factors, such as base station proximity, antenna downtilt angles, and foliage attenuation. Based on these empirical observations, route segments categorized as Poor or Fair represent high-risk zones for connectivity and should be prioritized for infrastructure enhancements, such as cellular repeater installation or targeted tower upgrades.

### 5.2. Lane Marking Evaluation

Reliable lane markings are a critical component of machine-readable roadway infrastructure [27,28]. They support lane-level localization and vehicle guidance, but their detectability can be reduced by degraded or missing markings, shadows, illumination changes, road construction, and repair activities [29]. Conventional field surveys provide direct engineering evidence, yet continuous network-scale inspection is resource-intensive [30,31]. A sample of qualitative lane detection results on different lane conditions is shown in Figure 6.

#### 5.2.1. Deep Learning-Based Detector

Lane markings were detected using Global Semantic Enhancement Network (GSENet) [32], configured with a ResNet-18 backbone and a feature pyramid network (FPN) [33]. A model checkpoint pretrained on the TuSimple dataset [34] was adopted. GSENet was selected for its global semantic enhancement strategy, which effectively handles challenging visual conditions—such as shadows, glare, and degraded lane markings—frequently encountered in rural video surveys.

During processing, input frames from the GoPro action camera were resized to 800 × 320 pixels. Candidate curves with a confidence score below 0.50 were discarded, after which non-maximum suppression (NMS) retained up to five lane candidates. To isolate the ego-lane boundaries relevant to the survey vehicle, the horizontal coordinate of each decoded curve at its lowest visible point was evaluated relative to the image center, assigning the closest candidate on each side as the left and right ego-lane boundaries. For each detected boundary, the lane-level positive-class Softmax score returned by GSENet was recorded as the detection confidence, reflecting the model’s certainty of the lane boundary’s existence. Finally, the decoded curves were transformed back to the original image resolution.

Because the adopted GSENet was pretrained on the TuSimple benchmark, an additional ground-truth experiment was conducted on a manually annotated subset to examine its transferability to the rural-road imagery collected in this study. A representative annotation is shown in Figure 7.

Two complementary metrics were employed. Point Coverage (PC) quantifies the fraction of valid ground-truth sampling positions for which a corresponding prediction is available:

(3) $$\mathrm{PC}=\frac{\sum_{i,b,k}I\left[P_{i,b}\left(y_{k}\right)\wedge G_{i,b}\left(y_{k}\right)\right]}{\sum_{i,b,k}G_{i,b}\left(y_{k}\right)}$$

where $G_{i,b}\left(y_{k}\right)$ and $P_{i,b}\left(y_{k}\right)$ are the ground-truth and predicted horizontal lane coordinates at sampling row $y_{k}$, respectively. PC measures prediction completeness rather than localization accuracy. Spatial agreement was evaluated per lane using IoU:

(4) $$\mathrm{IoU}=\frac{\left|P\cap G\right|}{\left|P\cup G\right|}$$

and the mean lane IoU was calculated as:

(5) $$\mathrm{mIoU}=\frac{1}{N}\sum_{i=1}^{N}{\mathrm{IoU}}_{i}$$

The same validation protocol was applied to BézierLaneNet [35], Global Association Network (GANet) [36], Ultra Fast Structure-Aware Deep Lane Detection (UFLD) [37], and GSENet. These models cover representative lane-detection paradigms. BézierLaneNet formulates lane boundaries using parametric Bézier curves, GANet performs keypoint-based global association, UFLD adopts an efficient row-wise classification strategy, and GSENet enhances lane representation through global semantic feature modeling. As summarized in Table 5, GSENet achieved a PC of 0.990 and a mean lane IoU of 0.854, which are the highest values among the four evaluated models. UFLD produced a comparable mean lane IoU of 0.852 but a lower PC of 0.957.

#### 5.2.2. Detection Indicators and Weighted Scoring

To evaluate lane marking detection across each 0.01-mile route segment *s*, three complementary indicators were calculated for each boundary b∈{L,R} (where *L* and *R* represent the left and right ego-lane boundaries, respectively).

The detection rate ($R_{b,s}$) measures the frequency with which boundary *b* is successfully detected within segment *s*:

(6) $$R_{b,s}=\frac{1}{n_{s}}\sum_{t\in s}I\left[c_{b}\left(t\right)>0\right]$$

where $n_{s}$ is the total number of synchronized frames in segment *s*, $c_{b}\left(t\right)$ is the GSENet confidence score for boundary *b* at frame *t*, and I[·] is an indicator function equal to 1 when a boundary is detected ($c_{b}\left(t\right)>0$) and 0 otherwise.

The conditional confidence ($C_{b,s}$) quantifies the average model certainty given that a valid detection occurred:

(7) $$C_{b,s}=\frac{\sum_{t\in s}c_{b}\left(t\right)I\left[c_{b}\left(t\right)>0\right]}{\sum_{t\in s}I\left[c_{b}\left(t\right)>0\right]}$$

The side-specific detectability index ($D_{b,s}$) represents the mean confidence across all frames, where non-detections are assigned a score of zero. Mathematically, $D_{b,s}$ is equivalent to the product of the detection rate and conditional confidence ($D_{b,s}=R_{b,s}\times C_{b,s}$):

(8) $$D_{b,s}=\frac{1}{n_{s}}\sum_{t\in s}c_{b}\left(t\right)$$

A weighted lane score was adopted to incorporate boundary-specific priorities into infrastructure screening. The rationale for emphasizing the left boundary is specific to rural two-way roads under right-hand traffic, where this boundary corresponds to the centerline separating opposing traffic. The MUTCD distinguishes this function from the roadway-delineation function of edge-line markings [38]. Research on centerline rumble strips also demonstrates the safety relevance of preventing encroachment into opposing traffic lanes on rural two-lane roads [39]. These sources motivate additional emphasis on centerline detectability but do not prescribe a numerical weighting ratio. In this work, a 2:1 left-to-right ratio was adopted as an engineering judgment for the present screening framework, which gives weights of 2/3 and 1/3 and assigns greater emphasis to the left boundary while preserving the contribution of the right boundary. The frame-level score and its segment-level average are defined as follows:

(9) $$w\left(t\right)=\frac{2}{3}c_{L}\left(t\right)+\frac{1}{3}c_{R}\left(t\right)$$

(10) $$D_{s}^{W}=\frac{1}{n_{s}}\sum_{t\in s}w\left(t\right)=\frac{2}{3}D_{L,s}+\frac{1}{3}D_{R,s}$$

The boundary weights represent an explicit aggregation assumption and are distinct from the AHP weights derived in Section 6. Their optimality and the robustness of the results to alternative ratios remain unverified. The centerline-based rationale is specific to the stated roadway configuration and requires reassessment where the left boundary serves a different function.

#### 5.2.3. Lane Marking Detection Results

As summarized in Table 6, under the adopted weighting scheme, the mean lane detectability score is 0.424. This value summarizes the two boundary indices with greater emphasis on the left boundary; the performance of the GSENet detector exhibited notable spatial variation between the left and right ego-lane boundaries. Overall, the weighted lane measure yielded an aggregate detection rate of 58.9% and a conditional confidence of 0.721. A comparative breakdown reveals that the left boundary was detected substantially more often than the right boundary, achieving a detection rate of 66.7% compared to 43.1%, respectively. Interestingly, despite this disparity in detection frequency, the conditional confidence—which measures model certainty when a detection successfully occurs—remained consistently high across both boundaries (0.717 for the left versus 0.730 for the right). This indicates that the lower mean detectability observed for the right boundary (0.315, compared to 0.478 on the left) is primarily driven by complete non-detections rather than low-confidence predictions. In rural survey environments, this asymmetry typically stems from obscured, worn, or unpaved road edges and encroaching roadside vegetation on the right shoulder, whereas centerlines on the left tend to be more continuously maintained.

Figure 8 visually corroborates these quantitative findings by illustrating the spatial distribution of lane detection confidence across the drive-test route. A direct comparison between Figure 8a and Figure 8b highlights a pronounced spatial asymmetry: while the left lane boundary maintains high to moderate confidence levels (dominant green-to-yellow regions) along the majority of the track, the right lane boundary exhibits severe, continuous dropouts to zero confidence (deep red bands)—most conspicuously along the entire western corridor and the eastern detour segment. This visual pattern confirms that the lower mean detectability observed on the right boundary (0.315) is driven by geographically continuous non-detections where right shoulder markings are eroded, unpaved, or obstructed by rural foliage. As illustrated in Figure 8c, applying the driver-side weighted scoring scheme successfully tempers right-edge dropout noise while accurately preserving critical spatial risk zones where both lane boundaries degrade simultaneously, offering a representative spatial map of usable ego-lane guidance.

### 5.3. Fast Assessments of Road Surface Conditions

To facilitate rapid and scalable evaluation of road surface conditions, this section presents a low-cost IRI regression model calibrated against field measurements from a high-speed AMES profiler which has been certified for repeatability and accuracy following TxDOT test method Tex-1001-S [40]. Furthermore, vehicle dynamic and kinematic indicators are integrated to capture multi-axis vehicle–road interactions, providing a holistic assessment of overall roadway driving stability.

#### 5.3.1. Low-Cost Rapid IRI Estimation

To establish a low-cost, scalable framework for rapid IRI evaluation, IMU and GPS telemetry were collected across 888 road segments (0.1-mile resolution) and calibrated against field profile data acquired via our high-speed AMES profiler. Pavement-induced vertical chassis vibrations were extracted using the Alternating Current (AC)-coupled/mean-subtracted root mean square ($RMS_{\mathrm{AC}}$) of *z*-axis acceleration over 1-second sliding windows (N=10). By subtracting the mean vertical component ($\bar{x}$), this AC-coupling process removes static 1g gravitational force and vehicle tilt, isolating dynamic vibrations directly induced by surface irregularities. To eliminate multicollinearity prior to model training, strongly correlated metrics—such as composite acceleration, which exhibited a high correlation coefficient with $RMS_{\mathrm{AC}}$ (r=0.85)—were excluded. The remaining independent variables capturing vehicle dynamics and road geometry derived exclusively from onboard IMU and GPS sensors are summarized in Table 7.

A multivariable Ordinary Least Squares (OLS) regression model was subsequently developed to predict section-level IRI from these sensor metrics. The model demonstrated satisfactory explanatory power and high statistical significance, achieving an $R^{2}$ of 0.702 (Adjusted $R^{2}=0.700$, *F*-statistic p≈0), indicating that approximately 70% of the variance in pavement roughness is captured by the low-cost sensor suite. As detailed in Table 8, $RMS_{\mathrm{AC}}$ served as the primary positive predictor of roughness (β=51.2204,p<0.001), while operating speed (β=−2.3512,p<0.001), cross slope (β=2.0536,p=0.005), and curve radius classifications controlled for dynamic energy scaling and geometric orientation effects. Conversely, longitudinal grade (p=0.122) and lateral load transfer ratio (p=0.082) were not statistically significant at the standard 95% confidence level (α=0.05), though LTR retained marginal significance at α=0.10. Model residual analysis confirmed normally distributed errors without systematic bias, validating that consumer-grade IMU and GPS sensors can effectively surrogate specialized profiling hardware for network-scale pavement monitoring.

#### 5.3.2. IRI Estimation Results

After training the regression model using field data from route 1, we test it on route 2; the IRI value was estimated at 0.01-miles interval. As shown in Table 9, lower IRI values indicate smoother and better-quality roads. Based on commonly adopted standards, pavements with IRI values below 95 in/mi are considered in Excellent condition, those from 95 to 125 are in Good condition, while those 125 to 173 are in Fair, and anything above 173 is considered in Poor condition. For the selected route, 75.7% fall under Excellent, 8.2% under Good, 6.1% are Fair, and 10.0% are classified as Poor.

Figure 9 presents the spatial distribution and statistical profile of the IRI across the evaluated test route 2. As illustrated in the spatial route heatmap (Figure 9a), the pavement along the primary corridor is predominantly smooth and well-maintained, as indicated by the extensive green segments along the loop. However, isolated high-roughness localized zones—rendered in yellow to dark red with values approaching or exceeding 173 in/mi—are visually discernible along specific straightaways and route transitions. The condition distribution histogram (Figure 9b) further corroborates these spatial observations, displaying a heavily right-skewed distribution where the vast majority of route segments fall into the Excellent condition category (<95 in/mi), with the primary bin containing over 3300 segments. Consequently, the median IRI of 65.7 in/mi reflects the high overall baseline pavement quality across the network, whereas the noticeably higher mean IRI of 90.8 in/mi demonstrates the disproportionate impact of localized high-roughness outliers extending into the Fair (125–173 in/mi) and Poor (>173 in/mi) threshold categories.

### 5.4. Vehicle Dynamic Safety and Kinematic Indicators

#### 5.4.1. Rollover Risk Assessment

Evaluating vehicle rollover risk via the Load Transfer Ratio (LTR)—hereafter designated as the primary rollover risk index (RRI) throughout this study—provides a direct physical measure of dynamic lateral stability and wheel unloading under real-world driving conditions. Rather than relying solely on raw lateral acceleration ($a_{y}$), the RRI quantifies the proportional shift in vertical wheel loads between the left ($F_{z,\mathrm{left}}$) and right ($F_{z,\mathrm{right}}$) tire tracks, which is modeled using measured lateral acceleration as:

(11) $$\mathrm{RRI}=\frac{F_{z,\mathrm{left}}-F_{z,\mathrm{right}}}{F_{z,\mathrm{left}}+F_{z,\mathrm{right}}}\approx \frac{2a_{y}h}{Tg}$$

where *h* denotes the height of the vehicle’s center of gravity, *T* represents the track width, and *g* is the acceleration due to gravity. By isolating lateral acceleration along the *y*-axis, the RRI identifies critical instability triggers, such as sharp evasive maneuvers, banked curve transitions, or asymmetric road surface anomalies (e.g., localized ruts or single-sided potholes). Because an RRI magnitude approaching ±1.0 signifies complete wheel lift-off and imminent rollover, this metric establishes a normalized, physics-based safety threshold that enables autonomous driving systems to trigger defensive path replanning or velocity reduction before reaching critical dynamic limits.

#### 5.4.2. Composite Acceleration

To comprehensively capture multidirectional dynamic disturbances, composed acceleration ($a_{\mathrm{comp}}$) is adopted as an orientation-invariant metric representing the total resultant acceleration vector imparted on the vehicle chassis. Computed as the Euclidean norm of the tri-axial acceleration components along the longitudinal ($a_{x}$), lateral ($a_{y}$), and vertical ($a_{z}$) axes:

(12) $$a_{\mathrm{comp}}=\sqrt{a_{x}^{2}+a_{y}^{2}+a_{z}^{2}}$$

This composite parameter ensures mathematical robustness against minor sensor mounting misalignments, vehicle pitch and roll, and road grade variations inherent in mobile deployments. Furthermore, severe localized road hazards—such as speed humps, bridge expansion joints, and deep potholes—exert coupled multi-axis impact forces on the vehicle body. By synthesizing these orthogonal components into a unified vector magnitude, composed acceleration provides a holistic evaluation of overall kinetic disturbance, aligning with standardized ride comfort frameworks [41] to effectively complement traditional single-axis roughness measures.

#### 5.4.3. Test Results

Figure 10 evaluates the spatial distribution of vehicle dynamic response indicators along the test route, comparing the RRI in Figure 10a and the CA in Figure 10b. As shown in both spatial heatmaps, the majority of the route maintains a low baseline excitation level (indicated by widespread green segments), corresponding to stable vehicle motion with RRI<0.04 and $\mathrm{CA}<2.0\;{m/s}^{2}$ along straight, well-paved road segments. However, prominent localized hotspots—rendered in orange to dark red—emerge concurrently across both indicators at specific roadway features. High RRI peaks (RRI>0.10) and elevated CA values ($\mathrm{CA}>4.5\;{m/s}^{2}$) are strongly co-located at sharp 90-degree turns, tight horizontal curves, and specific localized pavement depressions along the southern corridor. This high spatial alignment demonstrates that severe dynamic disturbances are primarily driven by the coupled effects of aggressive horizontal curvature and severe localized pavement roughness, serving as critical operational risk indicators where autonomous driving systems may experience increased vehicle instability or ride discomfort.

## 6. Aggregated Autonomous Vehicle Readiness Index

To synthesize the independent assessments established in preceding sections—cellular connectivity, visual lane detection confidence, and dynamic road surface conditions—into a single metric in terms of Analytic Hierarchy Process, appropriate contribution weights must be assigned to each indicator. For this purpose, AHP [42] is employed to derive criteria weights from structured expert judgment, yielding a consolidated metric termed the ARI.

AHP is a multi-criteria decision-making framework that decomposes complex evaluation problems into a structured hierarchy of goals, criteria, and sub-indicators [43]. Rather than relying on arbitrary weight assignments, AHP derives relative priorities through systematic pairwise comparisons. This approach is particularly advantageous for infrastructure readiness assessment because it integrates non-commensurate technical metrics—such as cellular signal strength, visual perception confidence, and vehicle dynamic excitations—into a unified, objective weighting scheme backed by mathematical verification of expert consistency.

### 6.1. Infrastructure Readiness Indicators

The evaluation framework is structured as the three-level hierarchy shown in Figure 11. The overarching goal, the ARI, is supported by three primary criteria: Communication (C), Lane Markings (LM), and Road Condition (RC), which are quantified by five operational indicators at the base level. Specifically, Communication is evaluated via Cellular Signal Strength, while Lane Marking is evaluated via Lane Marking Detection Confidence. Meanwhile, Road Condition encompasses three distinct indicators that capture different mechanisms of vehicle-road interaction: the IRI, the RRI, and CA. Because Communication and Lane Markings are each represented by a single indicator, those two indicators directly inherit the full priority weight of their parent criteria. In contrast, the three sub-indicators under Road Condition divide the overall priority weight assigned to that parent criterion.

Relative importance weights were elicited through a pairwise-comparison survey completed by a panel of domain contributors (N=40), comprising transportation engineering practitioners (n=2), senior autonomous vehicle faculty researchers (n=2), and specialized graduate research scholars in smart transportation infrastructure (n=36). While these derived weights establish a baseline preference vector for rural corridors, the framework is designed to be modular, enabling transportation agencies to recalibrate local priority vectors based on regional operational requirements. Each respondent evaluated six pairwise comparisons: three among the primary criteria (*C*, *LM*, and *RC*) and three among the sub-indicators under *RC* (*IRI*, *RRI*, and *CA*). Judgments were recorded using Saaty’s 1–9 fundamental scale [44], where 1 denotes equal importance, 3 moderate, 5 strong, 7 very strong, and 9 extreme importance, with intermediate even values (2, 4, 6, 8) available for refined assessments.

### 6.2. AHP Calculation and Consistency Evaluation

The derivation of indicator priorities from expert pairwise comparisons follows a four-stage mathematical procedure [44,45]. First, to aggregate individual comparison judgments from *N* experts into a single group comparison matrix $A=[a_{ij}]$, the geometric mean is applied across all expert responses:

(13) $$a_{ij}={\left(\prod_{k=1}^{N}a_{ij}^{\left(k\right)}\right)}^{1/N}$$

The geometric mean is mathematically required in group AHP aggregation to preserve the fundamental reciprocal property ($a_{ji}=1/a_{ij}$). Second, local priority weights $w={\left[w_{1},w_{2},\ldots ,w_{n}\right]}^{T}$ for each matrix are computed by solving the principal eigenvector problem:

(14) $$Aw=\lambda_{\max}w$$

where $\lambda_{\max}$ represents the principal eigenvalue of matrix *A*. The derived weight vector w is normalized such that $\sum_{i=1}^{n}w_{i}=1.0$. Third, to verify that expert judgments remain logically coherent, the Consistency Index (CI) is evaluated as:

(15) $$CI=\frac{\lambda_{\max}-n}{n-1}$$

where *n* denotes the order of the comparison matrix (n=3 for both the criteria and indicator matrices). The Consistency Ratio (CR) is subsequently calculated by comparing CI against the Random Index (RI) corresponding to matrix size *n* (RI=0.58 for n=3):

(16) $$CR=\frac{CI}{RI}$$

A threshold condition of CR≤0.10 is applied to confirm acceptable judgment consistency. Finally, global indicator weights ($w_{\mathrm{global}}$) are synthesized by multiplying the local weight of an indicator ($w_{\mathrm{local}}$) by the local weight of its parent criterion ($w_{\mathrm{criterion}}$):

(17) $$w_{\mathrm{global}}=w_{\mathrm{criterion}}\times w_{\mathrm{local}}$$

This hierarchical aggregation ensures that the sum of all global indicator weights equals 100%.

### 6.3. AHP Resulting Weights

The aggregated group comparison matrices, derived local priority weights, and consistency ratios are presented in Table 10. At the criteria level, Lane Markings received the highest priority at 56.0%, followed by Road Condition at 26.6% and Communication at 17.4%, achieving a highly consistent Group Consistency Ratio of 0.006. Within the Road Condition criterion, rollover risk index carried the largest weight at 42.9%, whereas IRI and CA contributed 28.7% and 28.4%, respectively, with a CR of 0.125.

While the criteria-level matrix demonstrates exceptional logical consistency (CR=0.006), well within Saaty’s standard threshold of 0.10, the Road Condition sub-criteria matrix yields a slightly elevated ratio (CR=0.125). Although CR≤0.10 is the conventional benchmark for acceptable consistency in AHP [46], previous applied studies have allowed moderately higher consistency ratios when working with real-world expert judgments, particularly when minor inconsistencies are difficult to avoid [47]. In this study, a relaxed threshold of CR≤0.15 was therefore adopted for the small 3×3 pairwise comparison matrices to accommodate minor inconsistencies inherent in multidisciplinary expert judgments. To assess whether this relaxation affects the conclusions, a sensitivity analysis was conducted by comparing the resulting priorities under CR≤0.10 (CR=0.086) and CR≤0.15 criterion (CR=0.125), as summarized in Table 11. Under CR≤0.10, the local weights for the RC sub-indicators were calculated as 42.9% for RRI, 30.2% for CA, and 26.9% for IRI (corresponding to global weights of 11.4%, 8.0%, and 7.2%, respectively). Compared to CR≤0.15—which yields local weights of 42.9% for RRI, 28.7% for IRI, and 28.4% for CA (corresponding to global weights of 11.4%, 7.6%, and 7.6%, respectively)—the primary priority weight for RRI remains unchanged at 11.4% globally, while IRI and CA exhibit minimal shifts of less than 0.4% globally. Such marginal deviations are acceptable in exploratory frameworks, particularly for low-dimensional comparison matrices (n=3) where a minor discrepancy in a single pairwise entry disproportionately impacts the consistency index due to the small Random Index (RI=0.58) and the lack of redundant comparisons. Furthermore, this localized dispersion reflects authentic variations in expert perspective regarding pavement roughness versus vehicle dynamic stability without altering the logical priority ranking of the sub-indicators. In future studies, expanding the expert sample size could further smooth individual judgment variances while preserving this core preference structure.

Local indicator weights were subsequently synthesized into global indicator weights by multiplying each local weight by its parent criterion weight, as summarized in Table 12. The final global weights reflect the overarching contribution of each operational metric, with Lane Marking Detection Confidence exerting the strongest influence (56.0%), followed by Cellular Signal Strength (17.4%), rollover risk index (11.4%), International Roughness Index (7.6%), and composite acceleration (7.6%). These weights are directly applied to the normalized indicator values across each 0.01-mile route segment to compute the spatial ARI.

### 6.4. Aggregated Spatial Mapping and Overall AV Readiness Assessment

To evaluate the holistic operational environment for AVs, the normalized sub-indicators were integrated using the AHP weight vector established in the previous subsection. Figure 12 presents the spatial distribution of the individual normalized metrics alongside the final aggregated ARI across the surveyed route 2.

As illustrated in Figure 12a,b, visual perception and wireless communication sub-indicators demonstrate substantial spatial variability. Because lane marking confidence holds the highest dominant priority (56.0%), spatial degradation in line visibility directly dictates the spatial trends observed in the overall ARI map (Right Panel). Specifically, the western corridor segment exhibits localized performance drops, defined here using a descriptive threshold of ARI < 0.40 (corresponding to the lower cluster of the empirical distribution in Figure 12f), depicted in red and orange hues, primarily driven by faded lane markings and inconsistent cellular network availability.

Specifically, the western corridor segment exhibits localized performance drops (ARI<0.40), depicted in red and orange hues, primarily driven by faded lane markings and inconsistent cellular network availability.

In Figure 12c–e, physical road infrastructure and dynamic vehicle response sub-indicators—namely rollover risk index (RRI), composite acceleration (CA), and pavement smoothness (IRI)—exhibit consistently high normalized values (∼1.0) across nearly the entire route length. This indicates that pavement macro-texture, ride comfort, and lateral vehicle dynamics do not pose severe operational bottlenecks under nominal driving conditions along this corridor.

The statistical distribution of segment-level readiness scores is summarized in the histogram in Figure 12f. The dataset exhibits a distinct bimodal distribution with an overall mean ARI score of 0.50 and a median of 0.43. The primary peak around ARI≈0.35–0.45 corresponds to segments constrained by suboptimal lane perception, whereas the secondary peak near ARI≈0.75–0.85 represents fully optimal segments where high-contrast markings, strong signal coverage, and smooth pavement align.

## 7. Conclusions and Future Work

This study presented a low-cost multi-sensor data fusion and multi-criteria decision-analysis framework for quantifying infrastructure preparedness for autonomous vehicles. By combining onboard optical cameras, high-precision GNSS/IMU platforms, and cellular network telemetry, the proposed Autonomous Vehicle Readiness Index (ARI) captures both physical infrastructure quality and digital Connectivity-Perception constraints into a unified spatially enabled metric. The experimental evaluation across the surveyed route yielded three primary insights: (1) Perception Dominance: Visual sensing infrastructure (lane marking visibility) serves as the single greatest driver of overall operational readiness, accounting for the majority of spatial readiness variance along the corridor. (2) Digital vs. Physical Disconnect: While physical road dynamics (roughness, composite accelerations, and rollover risk) remained consistently optimal across >90% of the route, digital connectivity and line detection experienced localized degradations. This emphasizes that AV ODD boundaries are constrained predominantly by perception and communication infrastructure rather than conventional pavement surface metrics. (3) Bimodal Operational Zones: The evaluated route demonstrated a clear bimodal distribution (Mean=0.50, Median=0.43), highlighting specific zones where infrastructure upgrades (e.g., remarking faded lanes and upgrading cellular roadside infrastructure) are needed prior to commercial AV deployment.

From a practical implementation perspective, the low-cost sensing suite is fully suitable for continuous data collection and near-real-time agency corridor screening; immediate onboard execution includes developing and deploying edge-platforms for true-real-time onboard execution with deep-learning processing loads and telemetry bandwidth adjustment. From a managerial and decision-making perspective, public transportation agencies can utilize the spatially localized ARI scores to shift from high-cost, reactive pavement resurfacing toward targeted, cost-effective interventions—such as prioritizing low-cost lane re-striping along structurally sound corridors showing severe perception degradation. Furthermore, AV fleet operators and dynamic routing engines can ingest these micro-scale ARI heatmaps to execute risk-based corridor geofencing, automatically adjusting automation fallback modes or re-routing vehicles before entering sub-threshold infrastructure zones.

While the presented methodology provides a low-cost baseline for AV route feasibility assessment, several directions remain open for future extension: (1) Empirical Validation of AV Performance: While this study focuses on infrastructure-side readiness assessment, future research will co-evaluate high- and low-ARI corridors with live autonomous test vehicles to empirically correlate ARI score gradients with real-time perception confidence, sensor fusion degradation, and vehicle disengagement rates. (2) Environmental and Weather Dynamics: Future iterations will incorporate dynamic, weather-dependent environmental degradation models (e.g., rain reflectance, night-time illumination, and fog scattering) to reflect real-time operational risk variations. (3) Edge Computing and Real-Time Telemetry: Integrating the processing pipeline into edge compute platforms on connected test vehicles could facilitate real-time streaming of ARI maps to dynamic routing engines and AV fleet management systems. (4) Vehicle-to-Infrastructure (V2I) Integration: Expanding the framework to account for dedicated short-range communications (DSRCs) and C-V2X direct communication infrastructure will provide a more comprehensive digital readiness score for Connected and Automated Vehicles.

## Figures and Tables

**Figure 1:**
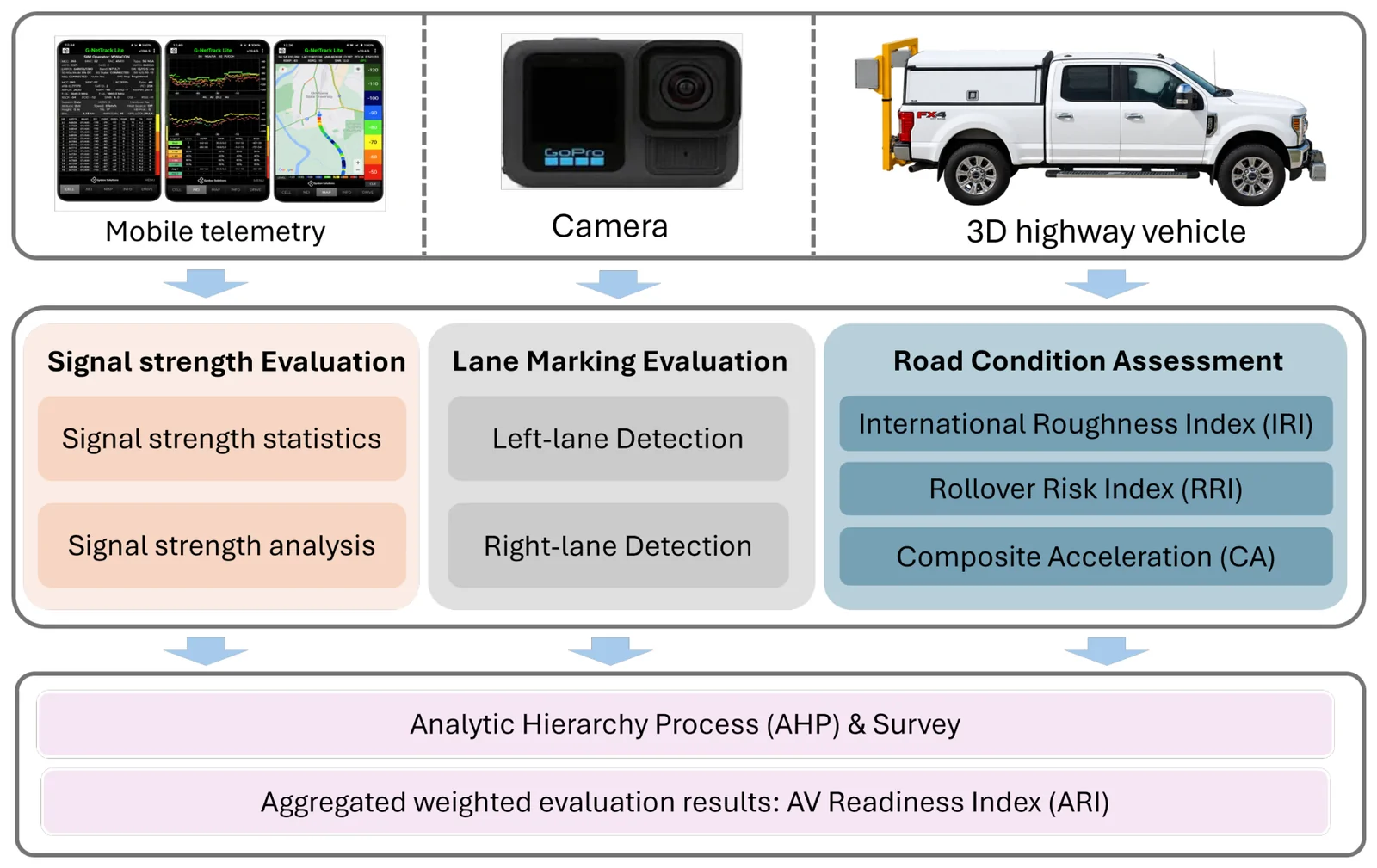
Proposed infrastructure preparedness evaluation framework.

**Figure 2:**
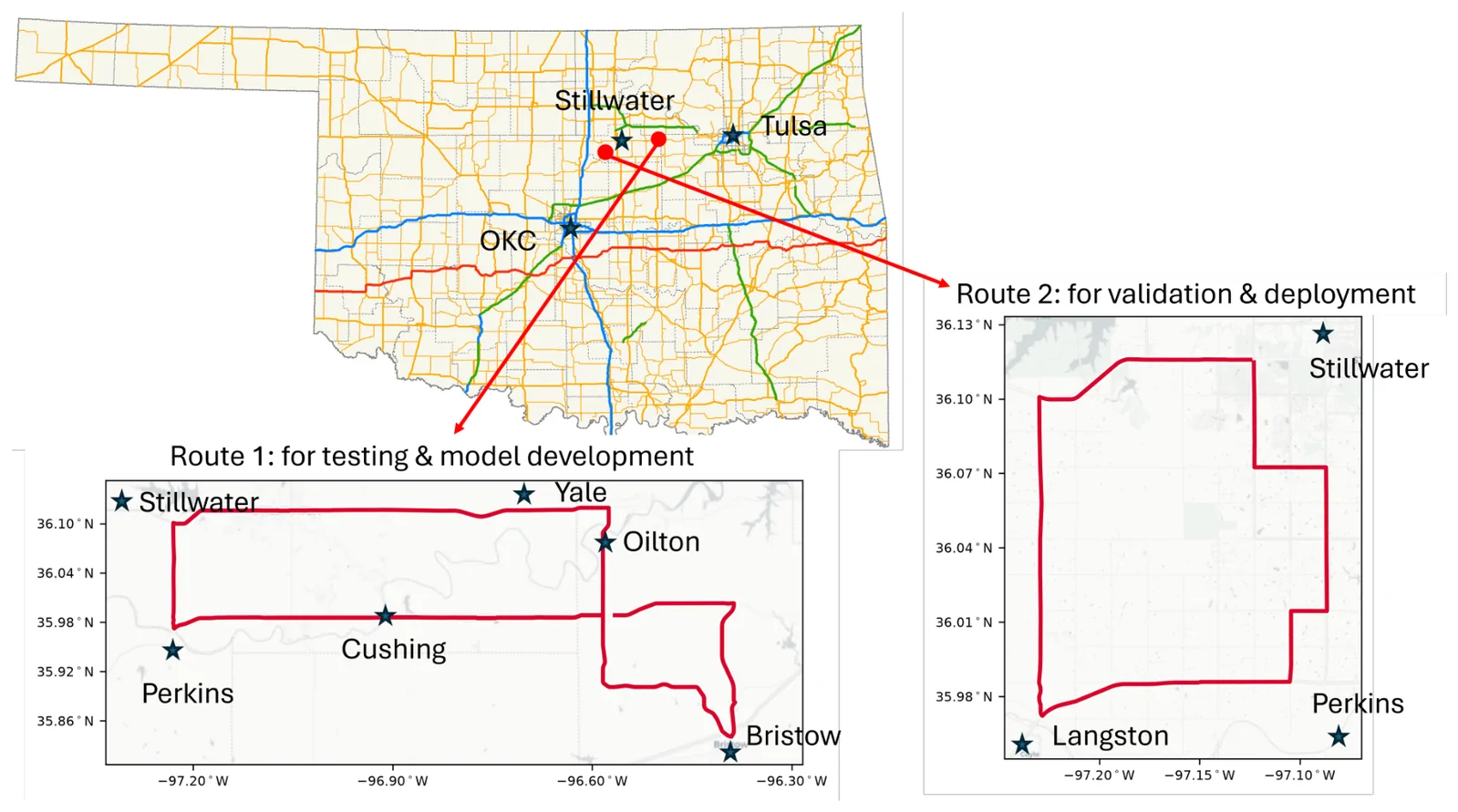
Selected rural routes for infrastructure preparedness evaluation. Stars represent major cities and towns within the study area.

**Figure 3:**
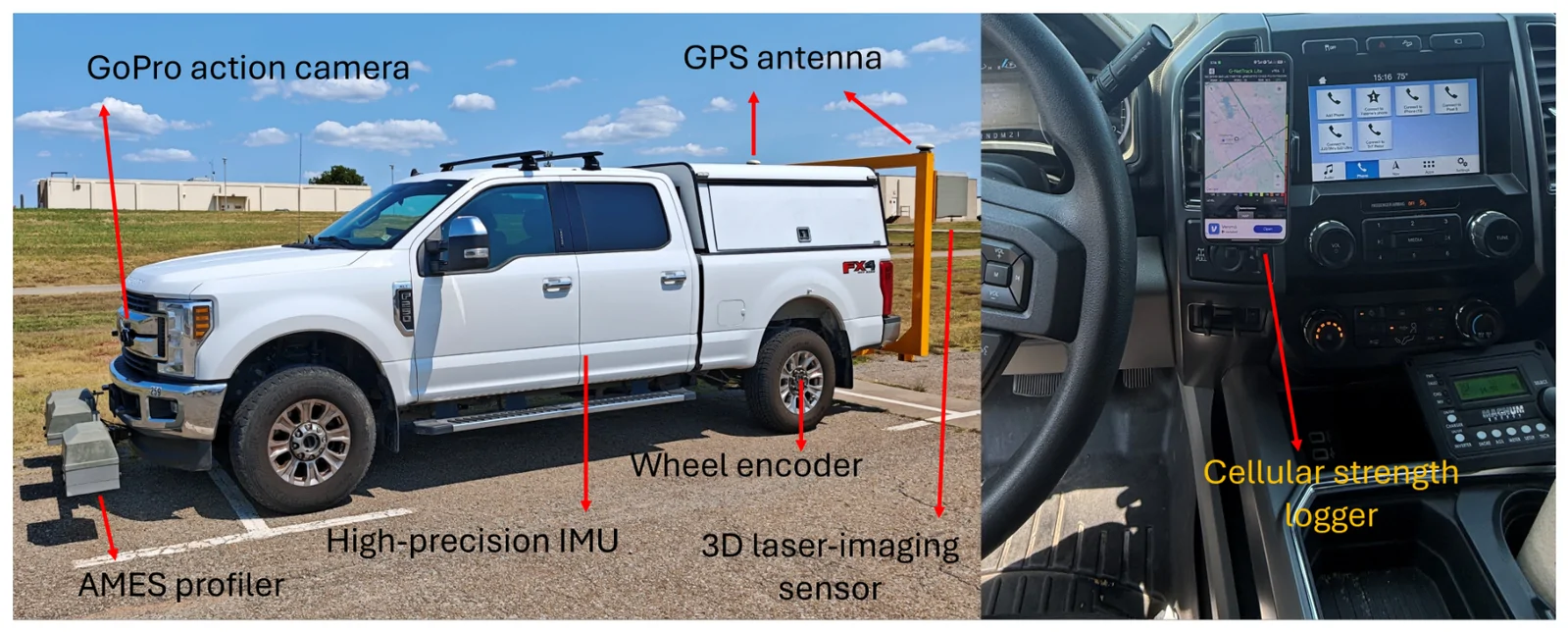
High-speed field testing platform.

**Figure 4:**
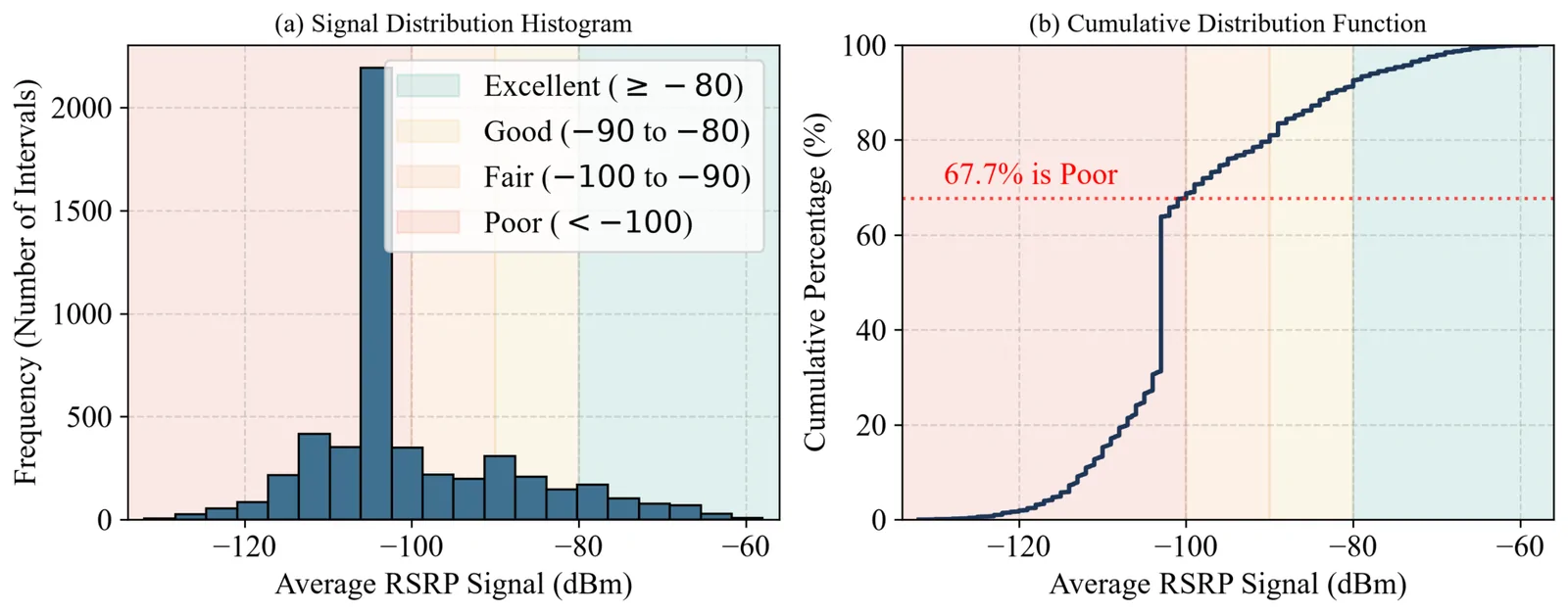
Spatial distribution of the average Reference Signal Received Power (RSRP) across the 0.01-mile evaluation intervals. (**a**) Histogram and (**b**) empirical Cumulative Distribution Function.

**Figure 5:**
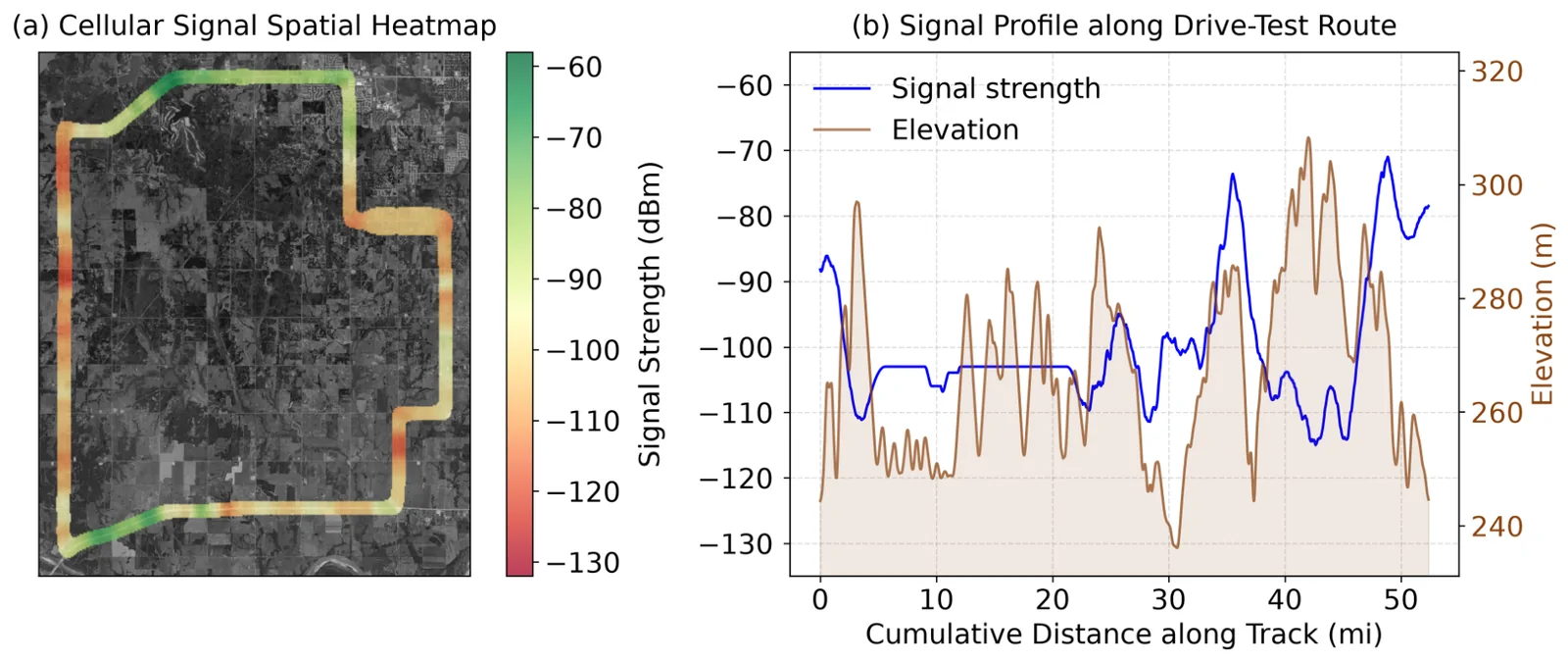
Cellular signal strength spatial heatmap and elevation profile. (**a**) Georeferenced signal strength heatmap. (**b**) Signal profile trend correlated with terrain elevation (m) across the trajectory.

**Figure 6:**
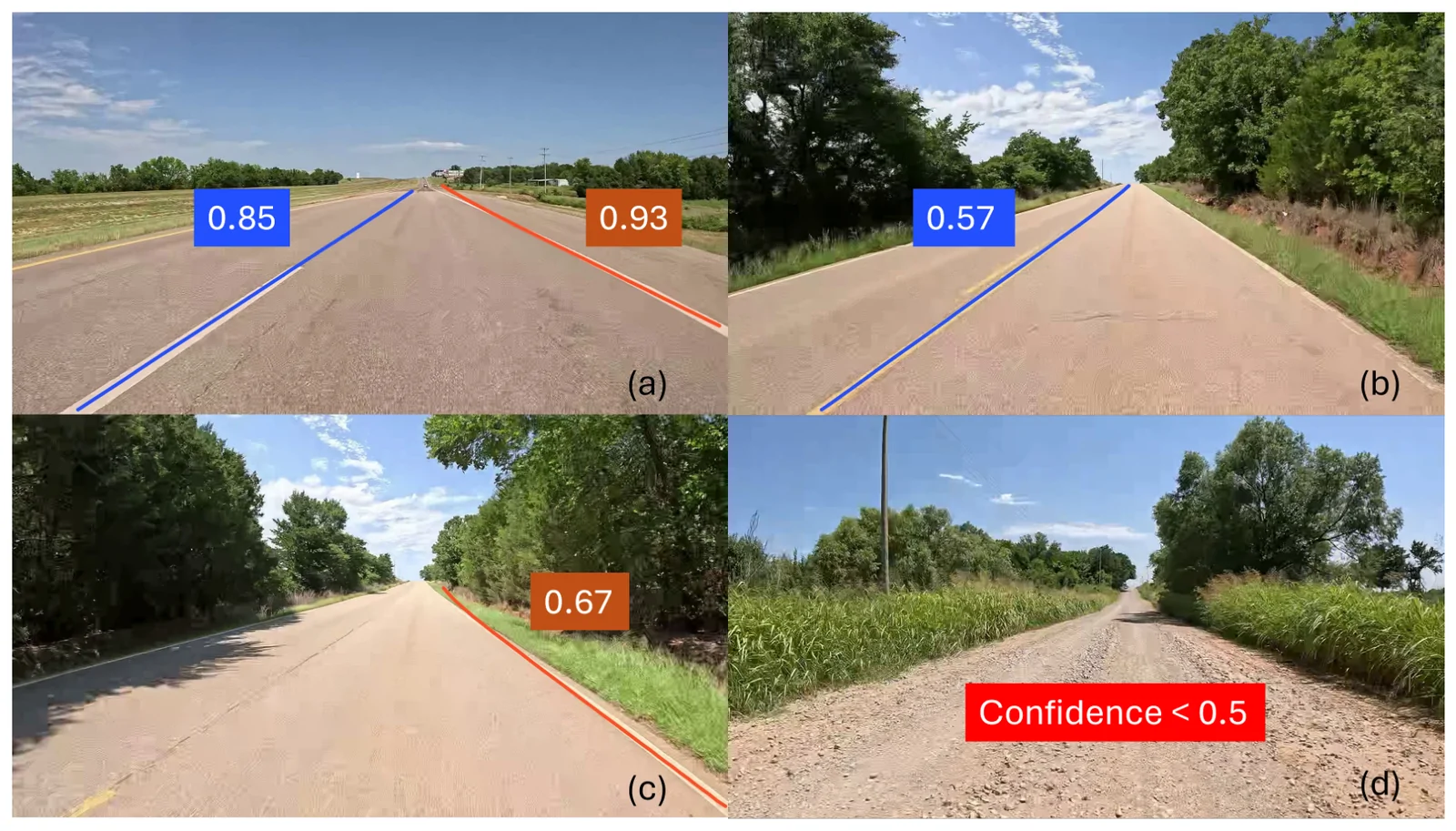
Qualitative examples of lane marking detection performance across varying rural road conditions: (**a**) Dual-boundary with high confidence on a well-marked road section; (**b**) single center-line under degraded shoulder conditions; (**c**) single right-edge under faded center-line conditions; (**d**) perception failure (Confidence < 0.5) on an unpaved gravel roadway.

**Figure 7:**
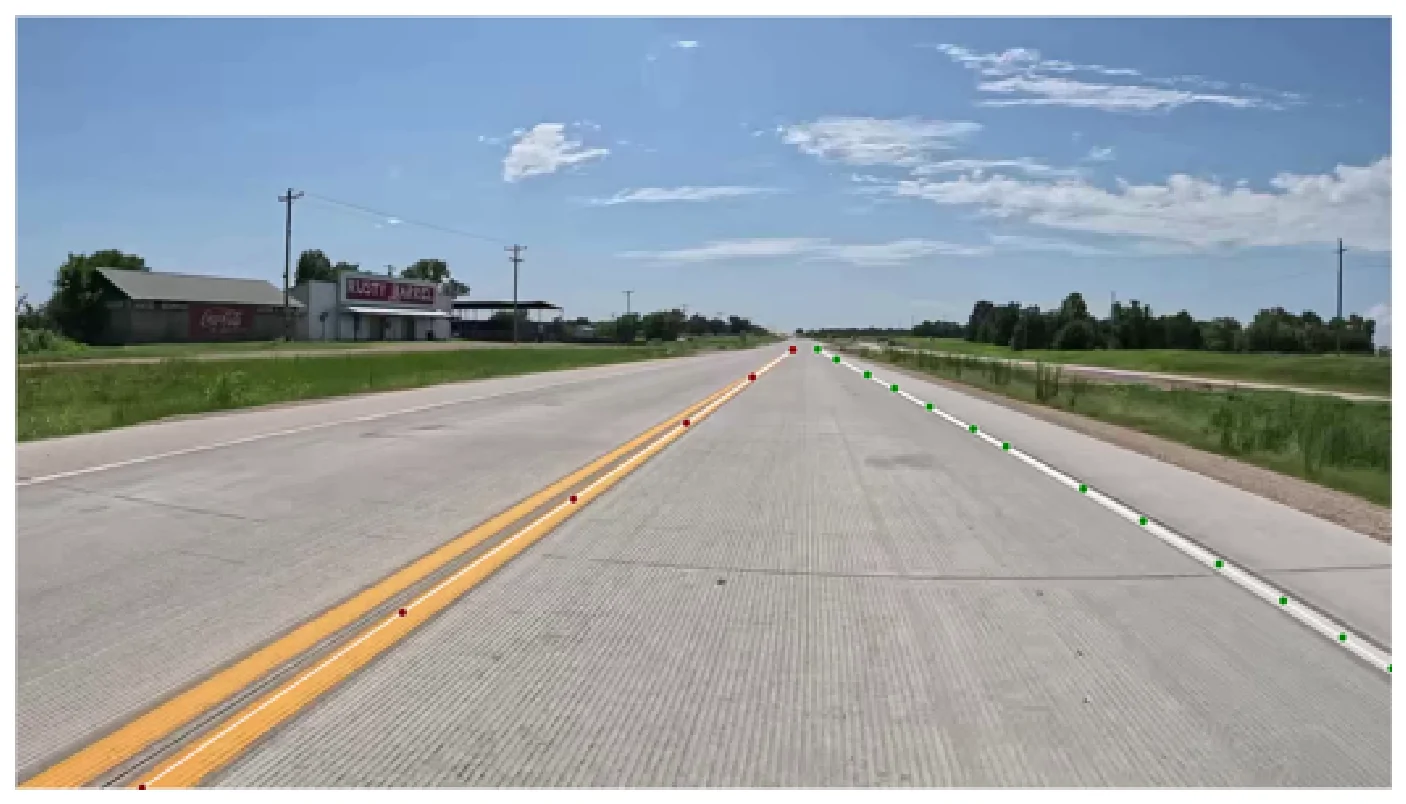
A representative annotation of ego-lane boundaries.

**Figure 8:**
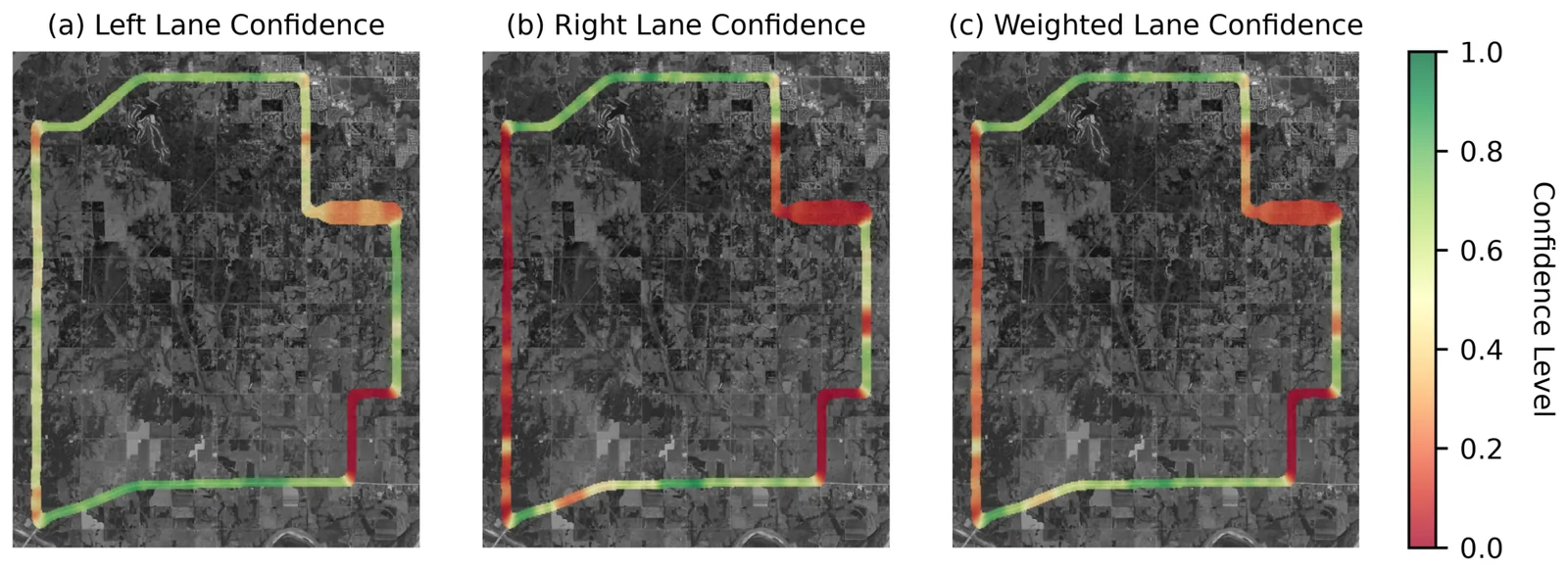
Spatial heatmaps of GSENet lane detection confidence levels mapped over satellite imagery. (**a**) Left lane boundary confidence. (**b**) Right lane boundary confidence. (**c**) Weighted lane confidence, providing a balanced spatial representation of ego-lane positioning availability.

**Figure 9:**
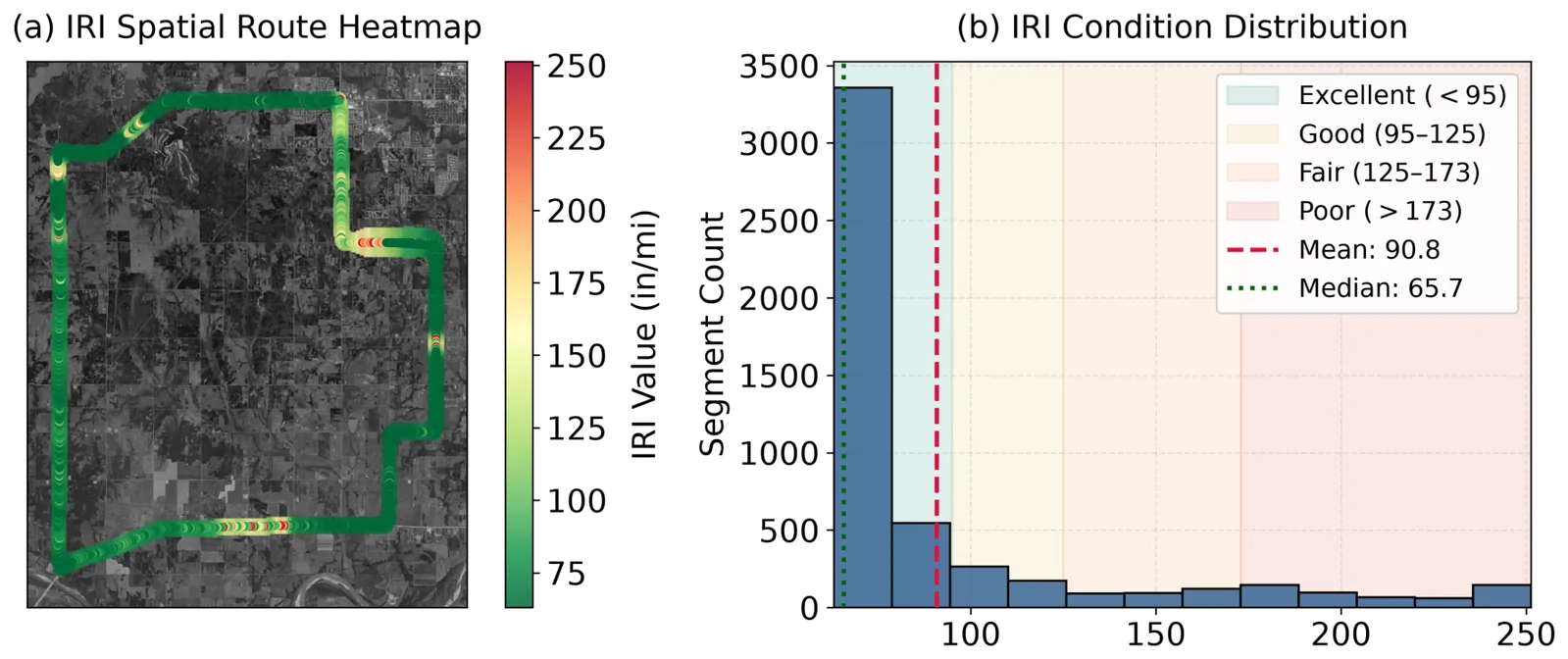
IRI estimation spatial heatmap (**a**) and distribution (**b**).

**Figure 10:**
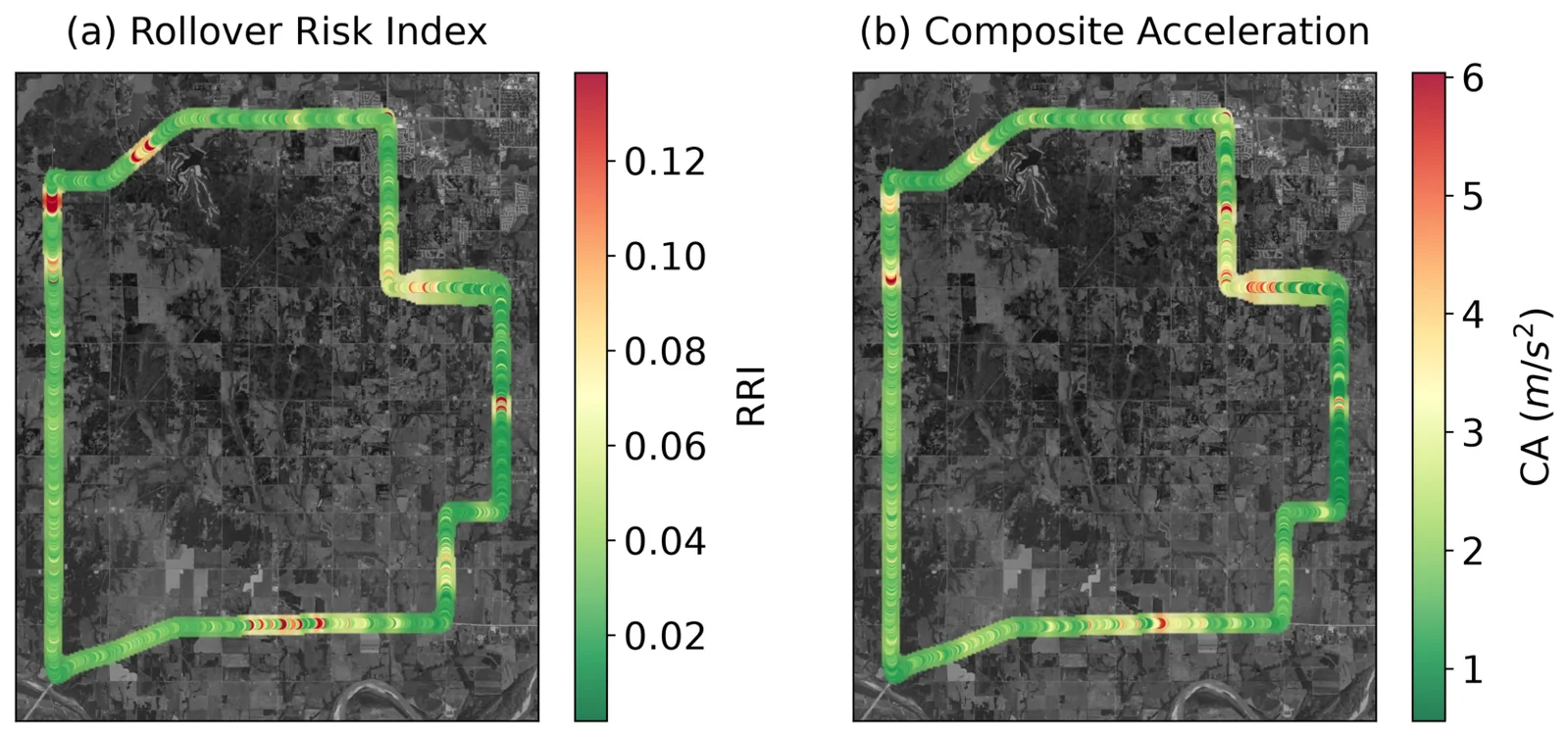
Spatial heatmaps of vehicle dynamics indicators along the test route: (**a**) RRI, quantifying rollover risk; (**b**) CA, measuring multi-axis dynamic excitations induced by combined road surface roughness and horizontal geometry.

**Figure 11:**
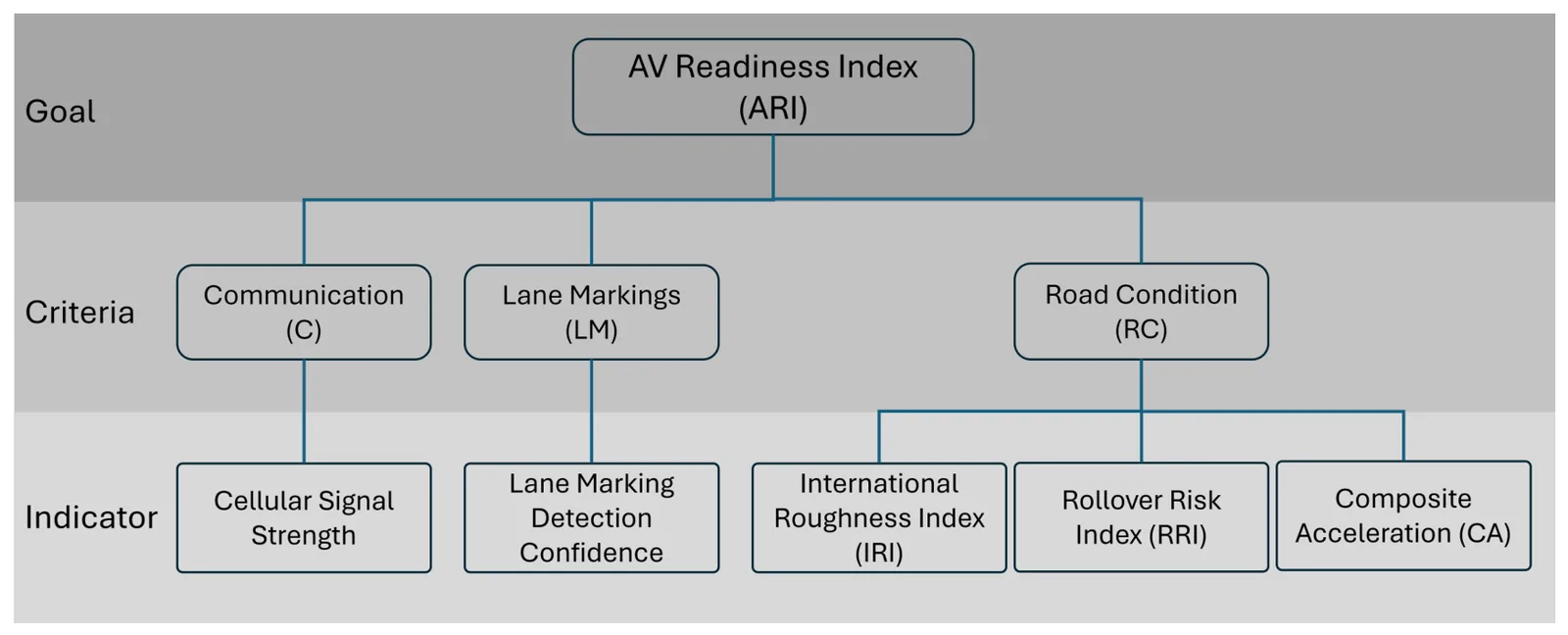
Hierarchy model for the AV Readiness Index (ARI).

**Figure 12:**
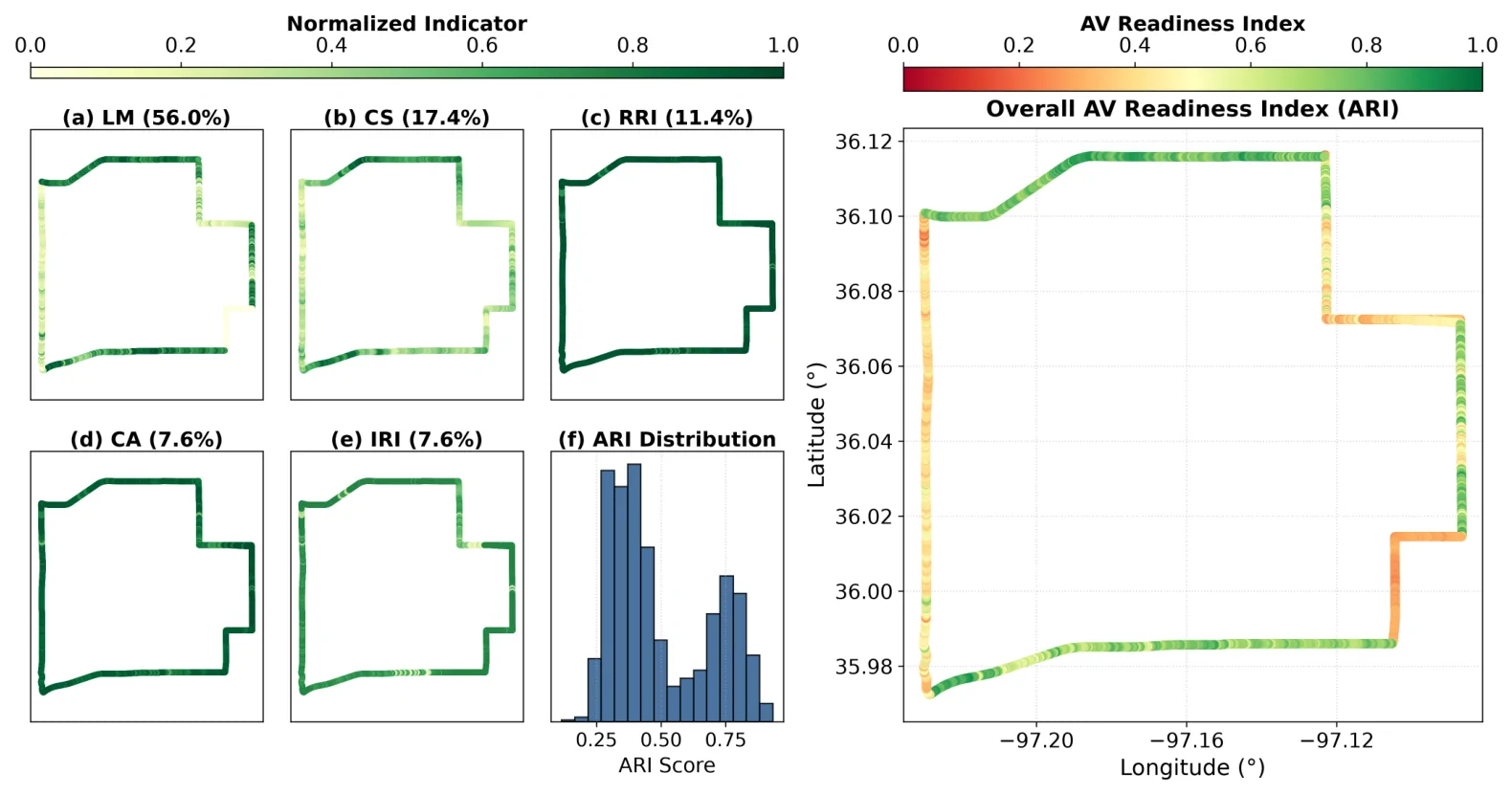
Multi-panel evaluation of autonomous vehicle readiness.

**Table 1:** Comparative analysis of AV infrastructure readiness frameworks.

Study	Target Scope	Data Source	Spatial Scale	Visual Perception	Digital Connectivity	Surface Kinematics
Khan et al. [15]	Urban (Macro)	Public data (Free)	City-wide	Qualitative	Policy level	Excluded
Kiattikomol et al. [24]	Urban Highways	Survey Rig (High cost)	Corridor	Optical Detection	5G Speed Test	Excluded
Alaka et al. [25]	Rural Counties	Census, DOT (Free)	County level	Agency Records	FCC Signal Maps	Excluded
Italiano et al. [26]	Highways	HD Maps (High cost)	Sub-segment	Signs, Markings	Static ODD Rules	Excluded
Proposed ARI	Rural, Tribal	Onboard Suite (Low cost)	Micro (0.01 mi)	DL Confidence	Cell Telemetry	RRI, CA, IRI

**Table 2:** Comparative summary of required hardware, software, and specifications across infrastructure studies.

Application Domain	Key Capabilities and Specifications	Hardware	Software and Protocols	Ref.
Roadway Departure and Friction	BSM telemetry parsing (ABS/ESC, yaw, wheel slip); low-friction risk mapping.	RSUs, Vehicle OBUs, Survey Vans	BSM parsers, CSW extensions, IHSDM CAD	[22]
Sight Distance and Auditing	3D point-cloud geometric auditing; line-of-sight and retroreflectivity.	Mobile LiDAR, retroreflectometers, RF analyzers	3D Voxel Raycasting, CV lane detection	[17]
Corridor Readiness and C-ITS	Segment HRI scoring (0–100%); ODD evaluation and disengagement alerts.	Dual-mode RSU (DSRC/CV2X), switches	IVIM protocols, ETSI C-ITS facility software	[26]
Smart City Cyber and Analytics	Low-latency V2X; fiber backhaul and real-time urban obstacle detection.	5G transceivers, fiber backhaul, cameras, edge servers	ML pedestrian vision, traffic analytics, 3D HD maps	[15,18]
On-Board Perception Nav.	Real-time 3D mapping, kinematic tracking, automated control.	LiDAR, mmWave radar, cameras, sonar, GPS/IMU	Deep CV (ResNet18), sensor fusion, AI planning	[16,20]
Device Security and Auditing	Statewide V2X health monitoring, SCMS security and cost–benefit analysis.	Signal Controllers (TSCs), SCMS servers	USDOT CV Manager, CIMMS monitoring	[23]
Socio-Economic Acceptance	Technology Readiness Index (TRI 2.0) and adoption analytics.	N/A	SmartPLS (SEM), discrete choice modeling	[19]
Rural and Tribal Infrastructure	ARI modeling	Smartphone, camera, GNSS, IMU	G-NetTrack Lite, GNSS/IMU software	This work

**Table 3:** Summary of indicator directionality, operational bounds, and normalization rules.

Indicator	Symbol	Direction	Raw Bounds	Optimal State (1.0)
Cellular Signal Strength	*C*	Benefit	[−130, −50] dBm	−50 dBm
Lane Marking Confidence	LM	Benefit	[0, 100]%	100%
International Roughness Index	IRI	Cost	[70, 250]	90
Rollover Risk Index	RRI	Cost	[0.0, 0.15]	0.0
Composite Acceleration	CA	Cost	[0.0, 0.6] *g*	0.0 *g*

**Table 4:** Reference Signal Received Power (RSRP)-based signal strength classification.

RSRP (dBm)	Signal Strength	Description
≥−80	Excellent	Strong signal with maximum data speeds.
−80 to −90	Good	Strong signal with good data speeds.
−90 to −100	Fair	Marginal signal: reliable speeds may occur, but dropouts are possible.
<−100	Poor	Weak signal: performance issues and data dropouts likely.

**Table 5:** Ground-truth validation of TuSimple-pretrained lane detectors on 300 manually annotated rural-road images.

Model	PC	Mean Lane IoU
BézierLaneNet	0.927	0.549
GANet	0.758	0.707
UFLD	0.957	0.852
GSENet	0.990	0.854

**Table 6:** Lane marking detection rate, conditional confidence, and mean detectability comparison.

Lane Measure	Assigned Weight	Detection Rate	Conditional Confidence	Mean Detectability
Left boundary	2/3	66.7%	0.717	0.478
Right boundary	1/3	43.1%	0.730	0.315
Weighted lane measure	2/3 + 1/3	58.9%	0.721	0.424

**Table 7:** Explanatory variables derived from IMU and GPS sensors for the multivariable OLS IRI estimation model.

Variable	Symbol	Source	Physical Description
Speed	*V*	GPS	Vehicle operating speed along track (ft/s)
Vertical Vibration	$RMS_{\mathrm{AC}}$	IMU ($a_{z}$)	AC-coupled (mean-subtracted) RMS of vertical acceleration
Load Transfer Ratio	LTR	IMU ($a_{y}$)	Estimated lateral load transfer ratio derived from lateral acceleration
Longitudinal Grade	$\theta_{\mathrm{pitch}}$	IMU	Vehicle pitch angle representing longitudinal slope (%)
Cross Slope	$\phi_{\mathrm{roll}}$	IMU	Vehicle roll angle representing transverse road tilt (%)
Curve Radius 1	$R_{1}$	GPS/IMU	Gentle curve/near-straight track segment baseline
Curve Radius 2	$R_{2}$	GPS/IMU	Moderate curve track segment
Curve Radius 3	$R_{3}$	GPS/IMU	Sharper curve track segment

**Table 8:** OLS regression results and coefficient statistics for section-level IRI prediction.

Variable	Coef.	Std. Err.	*t*-Stat	*p* > |*t*|	95% Conf. Interval	
					[0.025]	[0.975]
Intercept (const)	166.6821	22.963	7.259	0.000	121.614	211.750
Speed (ft/s)	−2.3512	0.092	−25.637	0.000	−2.531	−2.171
$RMS_{\mathrm{AC}}$	51.2204	1.516	33.798	0.000	48.246	54.195
LTR	126.3635	72.511	1.743	0.082	−15.951	268.678
Grade (%)	−0.7335	0.474	−1.548	0.122	−1.664	0.197
Cross Slope (%)	2.0536	0.737	2.786	0.005	0.607	3.500
Curve Radius 1 (Gentle)	−52.1941	8.025	−6.504	0.000	−67.945	−36.443
Curve Radius 2 (Moderate)	−31.3507	14.366	−2.182	0.029	−59.547	−3.154
Curve Radius 3 (Sharper)	30.9432	5.381	5.750	0.000	20.381	41.505

**Table 9:** Pavement condition classification based on IRI.

IRI Range (in/mi)	Condition	Description
<95	Excellent	Smooth, high-quality pavement with optimal ride comfort and negligible surface distress.
95–125	Good	Mostly smooth ride with minor surface irregularities; low vehicle vibration and minimal pavement wear.
125–173	Fair	Moderately rough ride; noticeable surface wear, minor cracking, or slight rutting causing mild vehicle vibration.
>173	Poor	Very rough ride with severe pavement distress (e.g., extensive cracking, rutting, or potholes) impacting ride safety and vehicle operating costs.

**Table 10:** Aggregated group comparison matrices and derived local priority weights.

Level	Element	vs. 1	vs. 2	vs. 3	Local Weight	Group *CR*
Criteria	Road Condition (RC)	1.000	1.403	0.517	26.6%	0.006
Criteria	Communication (C)	0.713	1.000	0.287	17.4%	
Criteria	Lane Markings (LM)	1.934	3.489	1.000	56.0%	
RC Indicators	Roughness (IRI)	1.000	0.458	1.474	28.7%	0.125
RC Indicators	Rollover Risk (RRI)	2.184	1.000	1.036	42.9%	
RC Indicators	Composite Acceleration (CA)	0.678	0.965	1.000	28.4%	

**Table 11:** Sensitivity analysis of local and global Road Condition indicator weights under CR≤0.10 and CR≤0.15.

Sub-Indicator	Parent	(CR≤0.10)		(CR≤0.15)	
		Local Weight	Global Weight	Local Weight	Global Weight
RRI	RC	42.9%	11.4%	42.9%	11.4%
CA	RC	30.2%	8.0%	28.4%	7.6%
IRI	RC	26.9%	7.2%	28.7%	7.6%

**Table 12:** Global indicator weights for the AV Readiness Index (ARI).

Indicator	Parent Criterion	Local Weight	Global Weight
Lane Marking Detection Confidence	LM	100.0%	56.0%
Cellular Signal Strength	C	100.0%	17.4%
Rollover Risk Index (RRI)	RC	42.9%	11.4%
International Roughness Index (IRI)	RC	28.7%	7.6%
Composite Acceleration (CA)	RC	28.4%	7.6%
Total			100.0%

## Data Availability

The data presented in this study are available on request from the corresponding author.
